# Knowledge and attitudes of health professionals towards pressure ulcers at a rehabilitation hospital: a cross-sectional study

**DOI:** 10.1186/s12912-016-0138-6

**Published:** 2016-03-05

**Authors:** Bayan Kaddourah, Amani K Abu-Shaheen, Mohamad Al-Tannir

**Affiliations:** Ambulatory Care Centre, Executive Administration of Nursing Services, King Fahad Medical City, Riyadh, Saudi Arabia; Research Center, King Fahad Medical City, P.O Box 59046, 11525 Riyadh, Saudi Arabia

**Keywords:** Attitudes, Knowledge, Pressure Ulcers, Quality indicator

## Abstract

**Background:**

Pressure ulcers are common conditions among hospitalized patients and impose substantial burden on patients and their caregivers. To assess the knowledge and attitudes of health professionals towards PUs prevention. Methods: A cross-sectional study was performed in the rehabilitation hospital at King Fahad Medical City, Riyadh, Saudi Arabia in 2014. The study population consisted of nurses, physical therapists, occupational therapists, and physical medicine rehabilitation physicians who have a minimum of at least one year of clinical practice. The survey that was created for use in this study consisted of demographic characteristics, Pressure Ulcers Knowledge Test and Staff Attitude Scale.

**Results:**

The survey was completed by 105 participants of the 120 total eligible staff. The mean knowledge score of correct answers from all participants was 34.1 ± 4.8 (71.5 %). Only 77(73.3 %) participants had a mean knowledge score of ≥ 70 %. The mean attitude score was 30.5 (56.5 %). The study revealed that age and profession factors had a significant relationship with participants’ mean knowledge of PUs prevention (P < 0.001), (P < 0.001) respectively. Moreover, 101 (98.1 %) participants are concerned about PUs prevention in their practices. While, 11 (10.7 %) of participants believe that PUs prevention is a time consuming procedure.

**Conclusions:**

The present study assessed the current knowledge and attitudes of health professionals regarding PUs prevention in an acute rehabilitation hospital. The majority of participants had an average level of knowledge and exhibited unsatisfactory attitudes towards PUs prevention. Increased health professionals awareness may improve their attitudes towards PUs prevention.

## Background

Pressure ulcers (PUs) are common conditions among hospitalized patients and impose substantial burden on patients and their caregivers. [1, 2]. Almost 1.7 million patients develop PUs per year [3]. Considerable variability in the incidence of PUs between developed and developing countries exists, with an estimated incidence rate of PUs of 8.3 % to 25.1 % in developed countries, and 2.1 % to 31.3 % in developing countries [4–6].

The incidence of PUs has become a universally known quality indicator in the hospital settings that the patients' quality of life, increases hospital expenses and has an adverse effect on achieving goals of care so much so that their occurrence reflects the quality of care [7, 8]. Thus, the treatment and prevention of ulcers should be considered as a priority, especially where patients are at high risk; such as patients in rehabilitation centers [9].

Lack of knowledge and skills in PUs prevention contributes substantially to the occurrence or deterioration of PUs [10]. Although evidence-based guidelines for the prevention of PUs have been developed extensively and have been supported globally, the problem is still widely spread in health care facilities around the world.

Knowledge, attitudes and skills are necessary to provide effective health care. Literature about the knowledge of health care providers towards PUs prevention is inconsistent. Some studies reveal that the overall knowledge is appropriate while others show that the knowledge about PUs is adequate [11, 12]. Also, despite the positive attitude towards PUs prevention [13], various studies have revealed a gap between theory and practice [3, 14, 15]. Within Saudi healthcare professionals, there is a scarcity of information regarding PUs. Considering the fact that better knowledge and attitudes result in better health care, the entire concerned disciplines should be aware, well informed and proficient at the clinical practice guidelines in order to reduce PUs.

Therefore, this study was undertaken to assess the knowledge and attitudes of the health professionals regarding PUs prevention in an acute rehabilitation hospital.

## Methods

### Study design

A cross-sectional study was conducted at the rehabilitation hospital at King Fahad Medical City (KFMC), Riyadh, Saudi Arabia in 2014.

### Study population

The study population comprised of all the nurses, physical therapists (PT), occupational therapists (OT), and physical medicine in rehabilitation (PMR) of both genders from the rehabilitation hospital that work directly with adult patients and have at least one year of clinical experience.

### Recruitment

Participants were invited for the study by an invitation letter given to the all eligible staff at the rehabilitation hospital. Along with the invitation letter, a copy of the questionnaire and a cover page describing the aim of the study, voluntary participation of the staff and contact information. A trained research coordinator handed the questionnaire to the participants and they were asked to answer the questionnaire and return it back to the research coordinator immediately.

### Survey tools

The PUs prevention survey developed for use in this study consisted of demographic characteristics that included gender, age, years of clinical experience, level of education, and profession. In addition, the study included a survey of the knowledge and attitudes of the participants about prevention of PUs.

Pressure Ulcer Knowledge Test (PUKT) was used to measure participants' level of knowledge and recommendations for PUs prevention. This test is based on the recommendations proposed in international guidelines and comprises of 47-items to examine the knowledge of participants' on PUs prevention, staging, and wound description. The participants were instructed to select an answer True, False or I Do Not Know. Each correct answer was considered one point. Correct answers corresponding to true assertions were answered with "T" and incorrect ones answered with "F". For incorrect or "NK" answers, the score was zero. The maximum score on the test was 47 and an average knowledge score of ≥ 70 % was considered satisfactory in this study. However, in the original study, participants who achieved 90 % or more of the correct answers were considered to have adequate knowledge [12]. The Staff Attitude Scale was used to obtain feedback on the attitudes of clinical staff regarding PUs prevention. The scale was designed by Moore and Price, it uses a 5-point scoring system ranging from strongly agree to strongly disagree [13]. To score, we assigned a numeric value to each response. For example, for "strongly disagree" =5, "disagree" = 4, and so on. However, questions 1, 6, 7, and 11 were reverse scored. For example, "strongly disagree" = 1, and so on. The scores ranged from 11 (most negative attitudes) to 55 (most positive attitudes).

### Ethical considerations

Ethical approval was obtained from the Institutional Review Board at KFMC. Participants who met the inclusion criteria were asked to participate in this study; those who agreed to take part gave written informed consent.

### Statistical analysis

All data was entered into and analyzed using SPSS version 22.0 software (SPSS Inc., Chicago, IL, USA). Categorical variables like profession, age group, level of education, and years of clinical experience were presented as numbers and percentages. Whereas, mean knowledge and attitude score of PUs prevention were expressed as Mean ± SD. Chi-square test, ANOVA and independent sample t-test were used as per condition of categories of variable to determine the mean knowledge score of PUs prevention with respect to the general characteristics of participants. P - Value of less than 0.05 was considered as statistically significant.

To improve power, we collapsed the Likert scale responses into two variables: combination of (“strongly agree” and “agree”) and combination of (“uncertain”, “disagree” and “strongly disagree”).

## Results

The survey was completed by 105 participants of the 120 total eligible staff who provide direct bedside care for patients from the rehabilitation hospital, and a response rate of 87.8 % was achieved. The demographic profile of respondents is presented in Table 1. The majority of participants were females 68 (64.8 %). Age ranged from 21 to 45 years, with a mean age of 37.5 ± 6.3 years. The majority had a bachelor’s degree 93 (88.6 %) with a mean years of clinical experience of 12.2 ± 7.3. Sixty-five (61.9 %) participants were nurses.

**Table 1:** Demographic Characteristics of Participants

Characteristics	Number of Participants (%)
Mean age years	37.5 ± 6.3
Median age years	35.0 [21-45]
Profession	
Nurse	65 (61.9)
OT	19 (18.1)
PT	14 (13.3)
PMR	7 (6.7)
Education level	
Diploma	2 (1.9)
Bachelor	93 (88.6)
Masters	3 (2.9)
Ph.D.	7 (6.7)
Mean years of clinical experience	12.2±7.3
Median years of clinical experience	10.2[1-25]

Data are presented either as mean (±SD), median [Min-Max] or actual numbers (%). OT: occupational therapists

PMR: Physical Medicine in Rehabilitation

PT: physical therapists

The possible score of the PUKT ranges from 0 to 47. The mean score of correct answers for all participants was 34.1(71.5 %) (SD = ±4.8, Min-Max = 18–44). Only 77(73.3 %) of participant had a mean percentage score of ≥ 70 %.

The statistical analysis of the participants’ demographics using ANOVA revealed that there was no significant relationship between participants’ means knowledge scores of PUs prevention and their education level, or years of clinical experience. In contrast, age and profession had a significant relationship with participants’ mean knowledge of PUs prevention (P < 0.001), (P < 0.001) respectively (Table 2).

**Table 2:** Mean knowledge score according to the geneal characteristics of personal profile

	Knowledge	p– value
	Mean (%)	
Overall	34.1(71.5)	---
Profession		<0.001
PMR	37.2 (79.3)	
Nursing	35.2 (75.0)	
OT	33.6 (71.7)	
PT	27.5 (58.5)	
Age (years)		< 0.001
≤30	37.7 (80.4)	
> 30	32.9 (70.0)	
Education level		0.092
Bachelor	33.8 (72.1)	
Diploma	36.0 (77.0)	
Masters	31.6 (63.0)	
Ph.D.	37.2 (79.3)	
Years of clinical experience		0.323
≤10	34.9 (74.3)	
>10	33.6 (71.6)	

Data are presented as mean and mean score percentage

OT: occupational therapists

PMR: Physical Medicine in Rehabilitation

PT: physical therapists

The lowest possible score (negative attitudes) in the attitudes, section was 11 with a highest possible score of 55. The participants demonstrated unsatisfactory attitudes towards PUs prevention. The mean attitudes score was 30.5 (56.5 %) (SD = ±4.8, Min-Max = 19–43).

The results presented in Table 3 revealed that 101 (98.1 %) participants are concerned about PUs prevention in their practices. While, 11 (10.7 %) participants reported that PUs prevention is a time consuming procedure. Furthermore, two (1.9 %) participants reported that PUs treatment is a greater priority than PUs prevention. Only seven (6.8 %) participants showed less interest in PUs prevention than other aspects of care.

**Table 3:** Participant’s attitudes towards pressure ulcer prevention

	Agreed *n* (%)	Disagreed *n* (%)
All patients are at potential risk of developing pressure ulcers	74 (71.8)	29 (28.2)
Pressure ulcer prevention is time consuming for me to carry out	11 (10.7)	92 (89.3)
In my opinion, patients tend not to get as many pressure ulcers nowadays	52 (50.5)	51 (49.5)
I do not need to concern myself with pressure ulcer prevention in my practice	2 (1.9)	101 (98.1)
Pressure ulcer treatment is a greater priority than pressure ulcer prevention	2 (1.9)	101 (98.1)
Continuous assessment of patients will give an accurate account of their pressure ulcer risk	101 (98.1)	2 (1.9)
Most pressure ulcers can be avoided	98 (95.1)	5 (4.9)
I am less interested in pressure ulcer prevention than other aspects of care	7 (6.8)	96 (93.2)
My clinical judgment is better than any pressure ulcer risk assessment tool available to me	8 (7.8)	95 (92.2)
In comparison with other areas of care, pressure ulcer prevention is a low priority for me	5 (4.9)	98 (95.1)
Pressure ulcer risk assessment should be regularly carried out on all patients during their stay in hospital	100 (97.1)	3 (2.9)

Data are presented as number and percentage.

Approximately 95.1 % of participants agreed that PUs could be avoided. Moreover, 100 (95.1 %) participants agreed that PUs risk assessment must be regularly carried out on all patients during their stay in hospital.

Our results indicate that PTs were the least interested among 'other' professionals in PUs prevention than other aspects of patient care and that PUs prevention is a low priority for them (p < 0.001), (p < 0.001) respectively. While nurses and OTs reported that their clinical judgment is better than PUs risk assessment in comparison to other areas of care (P = 0.041). Moreover, the study showed that there was no significant relationship between participants’ attitudes towards PUs prevention and their education level, years of clinical experience, and age (Table 4).

**Table 4:** General characteristics of personal profile affecting attitudes of participants

Items	Variables	Agree n (%)	Disagree n (%)	p– value
I am less interested in pressure ulcer prevention than other aspects of care	Profession			< 0.001
	Nursing	0(0.0)	64 (66.7)	
	OT	3 (42.9)	15 (15.6)	
	PMR	0(0.0)	7 (7.3)	
	PT	4 (57.1)	10 (10.4)	
My clinical judgment is better than any pressure ulcer risk assessment tool available to me	Profession			0.041
	Nursing	3 (37.5)	61 (64.2)	
	OT	3 (37.5)	15 (15.8)	
	PMR	2 (25)	5 (5.3)	
	PT	0(0.0)	14 (14.7)	
In comparison with other areas of care, pressure ulcer prevention is a low priority for me	Profession			< 0.001
	Nursing	0(0.0)	64 (65.3)	
	OT	1 (20)	17 (17.3)	
	PMR	0(0.0)	7 (7.1)	
	PT	4 (80)	10 (10.2)	

Data are presented as number and percentage

OT: occupational therapists

PMR: Physical Medicine in Rehabilitation

PT: physical therapists

## Discussion

Adequate knowledge about PUs prevention is crucial for health care staff. Such knowledge will help frame the decision of whether or not the patient is at higher risk and need prevention. It will also assist in knowing what type of prevention should be used, and how should it be practiced. Although scientific advances in health care guidelines and recommendations for PUs prevention are available, the problem is still widespread in health care facilities around the world. This study aimed to assess the current knowledge and attitudes of the health professionals towards PUs prevention in an acute rehabilitation hospital at KFMC.

The results of this study reveal that the knowledge of participants concerning PUs prevention was average. We used a ≥ 70 % cut-off point to identify participants having sufficient knowledge; at this cut-off point, 77(73.3 %) participants met the criterion. A greater cut-off point would have led to more participants being considered not having adequate knowledge, which highlights the need to update the health professional’s knowledge on current guidelines and recommendations for PUs prevention. The mean percentage score of correct answers was 71.5 % for all participants, 79.3 % for PMRs, 75.0 % for nurses, 71.7 % for OTs, and 58.5 % for PTs. It was noted from our results that nurses showed good mean percentage score of correct answers; this is especially important as good knowledge and practice of nurses have its own significant contribution for decreasing prevalence of PUs; because, even if the prevention of PUs is a multidisciplinary responsibility, usually nurses play a major role in PUs prevention. Likewise, a study conducted by Pieper et al, using the same knowledge scale showed that the mean percentage of correct answers by 75 intensive care unit nurses from two American hospitals was 71.3 % [16]. In the United States, a study of nurses from Montana used the preliminary version of the Pieper’s PUKT; found that the percentage of correct answers was 78 % [17]. While some studies have showed a good level of knowledge (70–80 %) among nurses [18–22], others have shown limited knowledge with only ≤ 50 % of nurses knowing half of the recommendations [1, 23, 24]. Similarly, a study conducted in Bangladesh indicated that the overall nurses’ knowledge on PUs prevention was found to be 57.79 % [25]. In Alexandria, Egypt, a study conducted by Enein et al. in one of the largest health insurance hospital showed that, the overall mean percentage score for nurses were below the minimum acceptable level [26], which would be explained by lack of learning resources for nurses to update their knowledge.

Younger age group participants significantly have higher mean of knowledge scores than older age group, however no significant relationship between participants’ mean of knowledge scores of PUs prevention and their education level or years of clinical experience was found. The findings are comparable to Pieper and Mott who did not find any association between educational level and knowledge [15, 27]. Knowledge on PUs prevention and treatment were known to be affected by certain individual and educational characteristics as revealed by studies on nurses and nursing students’ [11, 16].

Attitude is considered an essential individual characteristic as it determines individual expectations [28]. Ajzen and Fishbein stated in their study that an individual’s likelihood of carrying out a positive behavior is influenced by a positive attitude [29]. This statement is supported by Champion, Leach and Hicks who showed the positive impact of more positive attitudes on the quality of nursing practice [30, 31].

In this study, the participants demonstrated unsatisfactory attitudes towards PUs prevention with a mean attitude score of 30.5(56.5 %). A study conducted by Beeckman et al. [32], about the knowledge and attitudes of nurses towards PUsprevention in a Belgian hospital showed that the knowledge of nurses about PUs prevention was poor and only half of the nurses showed attitudes scores of equal to or greater than 75 % with a mean attitude score of 71.3 % [32]. In addition, our results showed that11 (10.7 %) of participants believe that PUs prevention is a time consuming procedure and among the health care professionals, nurses and OTs reported that that their clinical judgment is better in other areas of health care than use of available PUs risk assessment tool.

As PUs development during hospitalization is an important healthcare quality indicator, the adoption of a useful prevention system could eliminate the problem. Successful PUs prevention depends on health professionals’ knowledge and attitudes, especially for health professionals who provide direct patient care. It is necessary to understand that individual factors are known to influence a health care professionals’ knowledge, attitudes, and the use of evidence-based practice; hence, there is an urgent need for rehabilitation hospitals around Saudi Arabia to formulate strategies and relevant policies in order to combat the socio-economic burden of this problem. The results of this study could be used as a guide for making a strategic plan directed at adopting preventative measures for PUs that can improve the quality of health care services largely. We would recommend that a further follow up on research looking into ways to make the best use of education for the health professionals working in this critical area is carried out.

## Conclusions

The present study assessed the current knowledge and attitudes of health professionals regarding PUs prevention in an acute rehabilitation hospital. The majority of participants had an average level of knowledge about PUs prevention; they exhibited unsatisfactory attitudes towards PUs prevention. Increased health professionals awareness may improve their attitudes towards PUs prevention.

## Abbreviations

PUs: Pressure Ulcers
KFMC: King Fahad Medical City
PUKT: Pressure Ulcer Knowledge Test
OT: occupational therapists
PMR: Physical Medicine in Rehabilitation
PT: physical therapists
